# A phase I/II trial of fixed-dose stereotactic body radiotherapy with sequential or concurrent pembrolizumab in metastatic urothelial carcinoma: evaluation of safety and clinical and immunologic response

**DOI:** 10.1186/s12967-017-1251-3

**Published:** 2017-06-29

**Authors:** Nora Sundahl, Katrien De Wolf, Sylvie Rottey, Karel Decaestecker, Daan De Maeseneer, Annabel Meireson, Els Goetghebeur, Valérie Fonteyne, Sofie Verbeke, Pieter De Visschere, Dries Reynders, Mireille Van Gele, Lieve Brochez, Piet Ost

**Affiliations:** 1 Department of Radiation-Oncology and Experimental Cancer Research, University Hospital Ghent, De Pintelaan 185, 9000 Ghent, Belgium; 2 Immuno-Oncology Network Ghent (ION Ghent), Ghent, Belgium; 3 Department of Medical Oncology, University Hospital Ghent, De Pintelaan 185, 9000 Ghent, Belgium; 4 Cancer Research Institute Ghent (CRIG Ghent), Ghent, Belgium; 5 Department of Urology, University Hospital Ghent, De Pintelaan 185, 9000 Ghent, Belgium; 6 Department of Dermatology and Dermatology Research Unit, University Hospital Ghent, De Pintelaan 185, 9000 Ghent, Belgium; 7 Department of Applied Mathematics, Computer Science and Statistics and Stat-Gent CRESCENDO consortium, Ghent University, Krijgslaan 281 S9, 9000 Ghent, Belgium; 8 Department of Pathology, University Hospital Ghent, De Pintelaan 185, 9000 Ghent, Belgium; 9 Department of Radiology, University Hospital Ghent, De Pintelaan 185, 9000 Ghent, Belgium

**Keywords:** Urothelial cell carcinoma, Radiotherapy, Immunotherapy, Pembrolizumab, Immunomonitoring, Randomized clinical trial, SBRT, Anti-PD-1, Bladder cancer

## Abstract

**Background:**

Current first-line standard of therapy for metastatic urothelial carcinoma is platinum-based combination chemotherapy. Pembrolizumab in phase III has demonstrated a promising overall response rate of 21.1% in patients with progression or recurrence after platinum-based chemotherapy. Preclinical and clinical evidence suggests that radiotherapy has a systemic anti-cancer immune effect and can increase the level of PD-L1 and tumor infiltrating lymphocytes in the tumor microenvironment. These findings gave rise to the hypothesis that the combination of radiotherapy with anti-PD1 treatment could lead to a synergistic effect, hereby enhancing response rates.

**Methods:**

The phase I part will assess the dose limiting toxicity of the combination treatment of stereotactic body radiotherapy (SBRT) with four cycles of pembrolizumab (200 mg intravenously, every 3 weeks) in patients with metastatic urothelial carcinoma. The dose of both pembrolizumab and SBRT will be fixed, yet the patients will be randomized to receive SBRT either before the first cycle of pembrolizumab or before the third cycle of pembrolizumab. SBRT will be delivered (24 Gy in 3 fractions every other day) to the largest metastatic lesion. Secondary objectives include response rate according to RECIST v1.1 and immune related response criteria, progression-free survival and overall survival. The systemic immune effect triggered by the combination therapy will be monitored on various time points during the trial. The PD-L1/TIL status of the tumors will be analyzed via immunohistochemistry and response rates in the subgroups will be analyzed separately. A Simon’s two-stage optimum design is used to select the treatment arm associated with the best response rate and with acceptable toxicity to proceed to the phase II trial. In this phase, 13 additional patients will be accrued to receive study treatment.

**Discussion:**

The progress made in the field of immunotherapy has lead to promising breakthroughs in various solid malignancies. Unfortunately, the majority of patients do not respond. The current trial will shed light on the toxicity and potential anti-tumor activity of the combination of radiotherapy with anti-PD1 treatment and may identify potential new markers for response and resistance to therapy. *Trial registration* this trial is registered on clinicaltrials.gov (NCT02826564).

**Electronic supplementary material:**

The online version of this article (doi:10.1186/s12967-017-1251-3) contains supplementary material, which is available to authorized users.

## Background

Metastatic urothelial carcinoma remains a disease that is associated with few therapeutic options, poor prognosis and short-term survival. Worldwide, an estimated 429,800 of new cases of urinary bladder cancer and 165,100 deaths occurred in 2012 [1].

Cisplatin-containing combination therapy is standard-of-care for metastatic patients, with a median overall survival (OS) of 12.5–15 months and a long-term disease free survival in about 15% of patients [2–5]. Unfortunately, approximately 50% of patients are unfit for cisplatin-containing chemotherapy and may only be palliated with carboplatin-based regimens, without a statistically significant improvement in OS or progression-free survival (PFS) [2, 6].

Therapies blocking the PD-1/PD-L1 pathway (e.g. pembrolizumab, nivolumab and atezolizumab) have shown encouraging responses in patients with metastatic urothelial carcinoma, with an overall response rate of 15–26% [6–10]. This has resulted in the recent FDA approval of nivolumab and atezolizumab for the treatment of patients with locally advanced or metastatic urothelial carcinoma whose disease has worsened during or following platinum-containing chemotherapy, or within 12 months of receiving platinum-containing chemotherapy, either before (neoadjuvant) or after (adjuvant) surgical treatment. Patients responding to anti-PD-1/PD-L1 therapy often have tumors with elevated PD-1 and PD-L1 expression and are infiltrated by CD8+ cytotoxic tumor infiltrating lymphocytes (TIL) [8, 9, 11, 12]. These tumors are referred to as PD-L1+ TIL+.

Unfortunately, a substantial number of patients do not respond to anti-PD-1/PD-L1 therapy, often patients with low levels of tumor-infiltrating CD8+ T cells and no signs of T cell activation [12, 13]. These tumors are referred to as PD-L1− TIL−. It is hypothesized that in these non-responding patients, the tumor microenvironment might hinder T cell infiltration and induction of local endogenous immune responses.

Radiotherapy might increase response rates by creating a more permissive tumor microenvironment through increasing PD-L1 expression on tumor cells [14] and stimulating the accumulation and activation of CD8+ T cells [15], all markers for response. Preclinical evidence clearly indicates that combining radiotherapy with anti-PD-1 treatment increases the anti-tumoral activity of both treatments and even produces long-term survival [16]. On the one hand, radiotherapy might stimulate the induction of local endogenous immune responses by anti-PD-1 treatment. On the other hand, active immune stimulation by anti-PD-1 treatment within the tumor microenvironment might maximize radiation-induced antitumor immunity. These positive effects of radiotherapy are most often observed using high-dose per fraction radiotherapy (>5 Gy per fraction), which can be delivered safely in patients using stereotactic body radiotherapy (SBRT) [17–19].

Both anti-PD-1 treatment as well as SBRT (3 × 8 Gy) have shown to be safe when applied separately with grade 3–5 treatment related adverse events in 15% [10] and 11,7% [20] of patients respectively. Before the combination of drugs can be implemented in routine practice, the safety has to be established in a phase I trial as SBRT might increase the toxicity of anti-PD-1 treatment or the other way around. In addition, the timing of SBRT might influence the induction of antitumor immunity; yet this has not been thoroughly investigated [21]. Consequently, patients will be randomized into two arms with different timing of SBRT.

This trial uses a parallel phase I/II clinical trial design for combination therapies [22]. This design allows assessment of the safety and the efficacy of a combination therapy (pembrolizumab and SBRT) in a relatively small number of patients [22].

## Methods/design

### Objectives

The primary objective of the phase I trial is to evaluate the safety of the combination treatment and determine the SBRT-schedule associated with dose limiting toxicity (DLT) in <20% of patients.

The secondary objectives of the phase I trial are to assess the response rate according to the response evaluation criteria (RECIST) v1.1 and the immune related response criteria (irRC), local control, progression-free survival and overall survival. Exploratory endpoints include systemic immunologic responses and response rates in PD-L1− TIL− tumors.

### Trial design

**Fig. 1:**
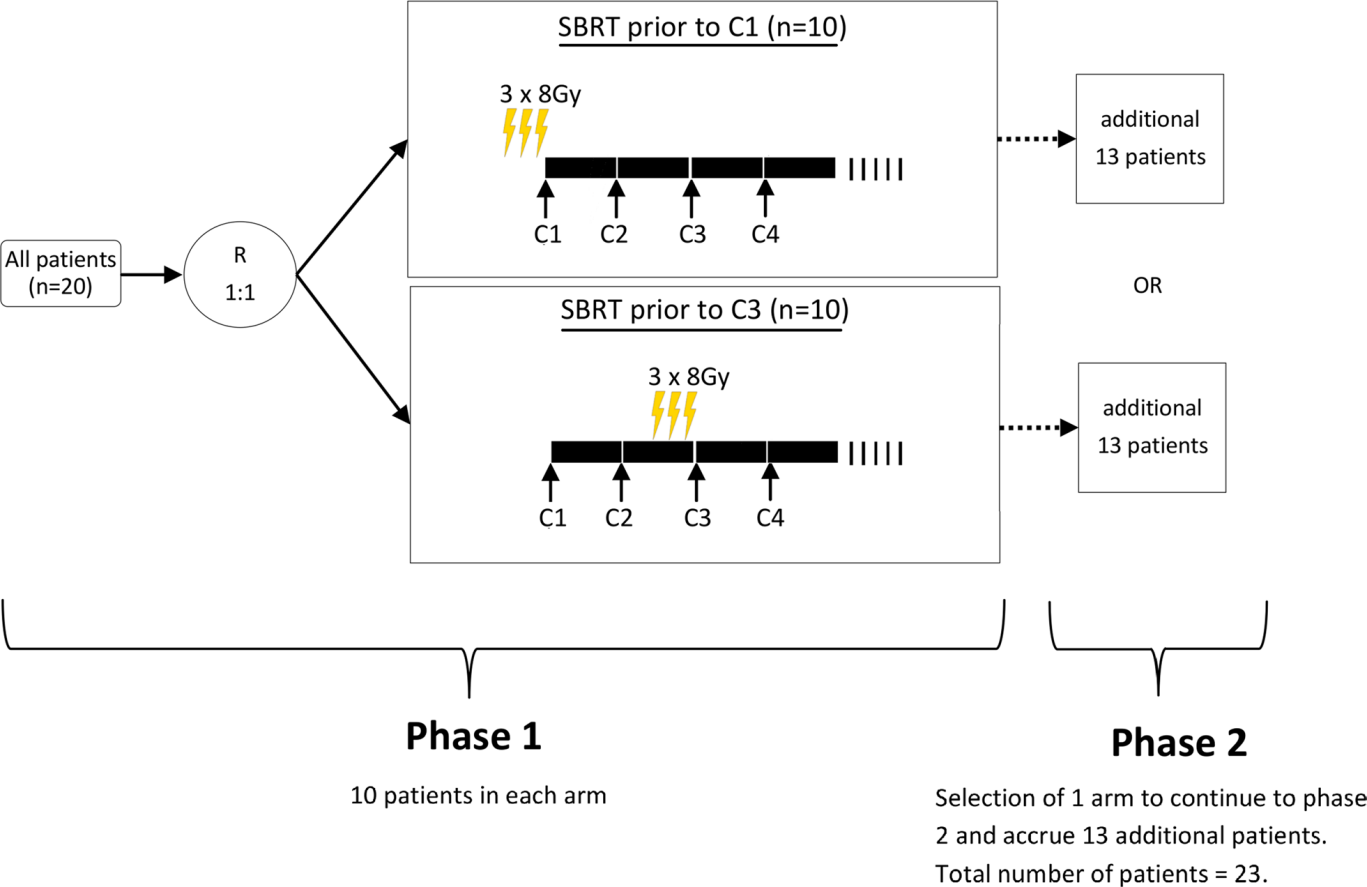
General scheme of the trial design

This trial uses a parallel phase I/II clinical trial design for combination therapies [22]. Pembrolizumab (200 mg, intravenously) will be administered every 3 weeks until disease progression or unacceptable toxicity. Patients will be randomized to receive SBRT either before the first cycle of pembrolizumab or before the third cycle of pembrolizumab. SBRT will be delivered (24 Gy in 3 fractions every other day) to the largest metastatic lesion that can be irradiated safely; the last fraction will be administered 1 day prior to the subsequent cycle of pembrolizumab. Figure 1 shows a general scheme of the trial design. A more detailed table of the trial enrolment, interventions and assessments can be found in the Additional file 1: Table S1.

### Outcome measures

#### Primary endpoint

- DLT occurring between the start of SBRT and 12 weeks after completion of SBRT. DLT will be assessed using the Common Terminology Criteria for Adverse Events (CTCAE) version 4.0. DLT should be, completely or in part, related to trial therapy. The following events will be considered as DLT:
  - any grade 3–5 metabolic or hematological toxicity that is related, probably related or possibly related to pembrolizumab or SBRT.
  - any grade 3–5 non-hematological toxicity that is related, probably related or possibly related to SBRT.

#### Secondary endpoints

- Objective response rate in the non-irradiated metastases as determined by RECIST v1.1 [23]. Response rate will be defined as the percentage of subjects achieving either a complete or a partial response at 12 weeks after the start of pembrolizumab. During treatment all patients will undergo a CT of the chest, abdomen and pelvis every 9 weeks or sooner if clinically indicated.
- Objective response rate in the non-irradiated metastases as determined by irRC [24] at 12 weeks after the start of pembrolizumab.
- Local control defined as the time between local irradiation and the moment the irradiated lesion shows an increase in size of ≥20%, according to the RECIST v1.1, confirmed by a consecutive assessment at least 4 weeks after first documentation.
- PFS defined as the time from inclusion to documented disease progression according to irRC or death from any cause.
- OS defined as the time from inclusion to death from any cause.

#### Exploratory endpoints

- Response rate according to expression of PD-L1. PD-L1 expression will be determined by immunohistochemistry in QualTek Molecular Laboratories on an archival or newly obtained tissue sample of the tumor or metastatic lesion.
- Immunologic response in peripheral blood samples analyzed via fluorescence-activated cell sorting (FACS) phenotyping, ultra-performance liquid chromatography (UPLC) and enzyme-linked immunesorbent assay (ELISA).

### Study population

#### Inclusion criteria

- Be willing and able to provide written informed consent/assent for the trial.
- Be ≥18 years of age on day of signing informed consent.
- Have at least two measurable lesions according to RECIST v1.1.
- Have had any prior treatment more than 2 weeks prior to study day 1, treatment naïve patients are allowed.
- Histological confirmed diagnosis of urothelial carcinoma.
- Be willing to provide tissue from a newly obtained core or excisional biopsy of a tumor lesion. *Newly*-*obtained is defined as a specimen obtained up to 6* *weeks (42* *days) prior to initiation of treatment on day 1. Subjects for whom newly*-*obtained samples cannot be provided (e.g. inaccessible or subject safety concern) may submit an archived specimen only upon agreement from the sponsor.*
- Have a performance status of 0 or 1 on the ECOG performance scale.
- Demonstrate adequate organ function as defined in Table 1, all screening labs should be performed within 10 days before treatment initiation.

**Table 1:** Adequate organ function laboratory values

System	Laboratory value
Hematological	
Absolute neutrophil count	≥1500/mcL
Platelets	≥100,000/mcL
Hemoglobin	≥9 g/dL or ≥5.6 mmol/L without transfusion or EPO dependency (within 7 days of assessment)
Renal	
Serum creatinine *OR* Measured or calculated^a^ creatinine clearance	≤1.5 X upper limit of normal (ULN) *OR* ≥60 mL/min for subject with creatinine levels >1.5 X institutional ULN
Hepatic	
Serum total bilirubin	≤1.5 X ULN *OR*
	Direct bilirubin ≤ULN for subjects with total bilirubin levels >1.5 ULN
AST (SGOT) and ALT (SGPT)	≤2.5 X ULN *OR* ≤5 X ULN for subjects with liver metastases
Albumin	≥25 g/L
Coagulation	
International normalized ratio (INR) or prothrombin time (PT) Activated partial thromboplastin time (aPTT)	≤1.5 X ULN unless subject is receiving anticoagulant therapy as long as PT or PTT is within therapeutic range of intended use of anticoagulants ≤1.5 X ULN unless subject is receiving anticoagulant therapy as long as PT or PTT is within therapeutic range of intended use of anticoagulants

^a^Creatinine clearance should be calculated per institutional standard
- Female subjects of childbearing potential should have a negative urine or serum pregnancy within 72 h prior to receiving the first dose of study medication. If the urine test is positive or cannot be confirmed as negative, a serum pregnancy test will be required.
- Female subjects of childbearing potential must be willing to use an adequate method of contraception for the course of the study through 120 days after the last dose of study medication. *Note: Abstinence is acceptable if this is the usual lifestyle and preferred contraception for the subject.*
- Male subjects of childbearing potential must agree to use an adequate method of contraception starting with the first dose of study therapy through 120 days after the last dose of study therapy. *Note: Abstinence is acceptable if this is the usual lifestyle and preferred contraception for the subject.*
- The subject is currently not participating or receiving study therapy and has not participated in a study of an investigational agent and received study therapy or used an investigational device within 4 weeks of the first dose of treatment.
- No prior radiotherapy interfering with SBRT.
- No diagnosis of immunodeficiency and no systemic steroid therapy or any other form of immunosuppressive therapy within 7 days prior to the first dose of trial treatment.
- No history of active tuberculosis.
- No hypersensitivity to pembrolizumab or any of its excipients.
- Has not received any prior anti-cancer monoclonal antibody within 4 weeks prior to study day 1 or has not recovered (i.e. ≥grade 1 or at baseline) from adverse events due to agents administered more than 4 weeks earlier.
- No prior chemotherapy, targeted small molecule therapy, or radiation therapy within 2 weeks prior to study day 1 or no recovering (i.e. ≤grade 1 or at baseline) from adverse events due to a previously administered agent.
  - Note: Subjects with ≤grade 2 neuropathy are an exception to this criterion and may qualify for the study.
  - Note: If subject received major surgery, they must have recovered adequately from the toxicity and/or complications from the intervention prior to starting therapy.
- No additional malignancy that is progressing or requires active treatment. Exceptions include basal cell carcinoma of the skin or squamous cell carcinoma of the skin that has undergone potentially curative therapy or in situ cervical cancer.
- No active central nervous system metastases and/or carcinomatous meningitis. Subjects with previously treated brain metastases may participate provided they are stable (without evidence of progression by imaging for at least 4 weeks prior to the first dose of trial treatment and any neurologic symptoms have returned to baseline), have no evidence of new or enlarging brain metastases, and are not using steroids for at least 7 days prior to trial treatment. This exception does not include carcinomatous meningitis which is excluded regardless of clinical stability.
- No active autoimmune disease that has required systemic treatment in the past 2 years (i.e. with use of disease modifying agents, corticosteroids or immunosuppressive drugs). Replacement therapy (e.g. thyroxine, insulin, or physiologic corticosteroid replacement therapy for adrenal or pituitary insufficiency, etc.) is not considered a form of systemic treatment.
- No known history of, or any evidence of active, non-infectious pneumonitis.
- No active infection requiring systemic therapy.
- No history or current evidence of any condition, therapy, or laboratory abnormality that might confound the results of the trial, interfere with the subject’s participation for the full duration of the trial, or is not in the best interest of the subject to participate, in the opinion of the treating investigator.
- No known psychiatric or substance abuse disorders that would interfere with cooperation with the requirements of the trial.
- Is not pregnant or breastfeeding, or expecting to conceive or father children within the projected duration of the trial, starting with the pre-screening or screening visit through 120 days after the last dose of trial treatment.
- Has not received prior therapy with an anti-PD-1, anti-PD-L1, or anti-PD-L2 agent.
- No known history of human immunodeficiency virus.
- No known active hepatitis B or hepatitis C.
- Subjects should not have received a live vaccine within 30 days of planned start of study therapy.

### Enrolment, randomization and evaluation

Patients must be restaged within 4 weeks prior to randomization with CT scans of the chest, abdomen and small pelvis. Patients will be enrolled by NS; they will then be randomly allocated to treatment arm A or B. The allocation sequence will be generated by EG and DR. Randomization will find place according to the time-to-event continual reassessment method (TITE-CRM) [25] with a variable length block design. This entails that prior to randomization of the next block of patients, the posterior probability of the toxicity at that time point will be determined. If the chance that the posterior probability exceeds 0.2 is more than 0.7, the treatment arm will be closed. No blinding will find place.

### Intervention

#### SBRT

A total dose of 24 Gy will be delivered to one lesion in 3 fractions with image-guided treatment verification and fractions will be separated >48 and <96 h.

All patients will receive a CT in supine position with 3 mm CT slice thickness through the tumor site. The planning simulation should cover the target and all organs at risk. A typical scan length should extend at least 10 cm superior and inferior beyond the treatment field borders. Support devices to increase patient comfort will be chosen depending on the tumor localization. Lung and liver tumor sites will be simulated with 4D-CT, taking into account breathing. The isocenter will be determined on the CT-simulator with marking of laser lines on the patient. Imaging data will be transferred to the treatment planning system. For all lesions, the gross target volume (GTV) will be defined as all visible tumor by combining iconographic and metabolic information. No additional margin will be added for microscopic spread of disease. The GTV will be expanded with 2–5 mm to the planning target volume (PTV) to account for organ motion and setup error. Margins depend on the site irradiated, with 2 mm margins for bony lesions and 5 mm for other sites. The type of organ at risk delineated depends on the localization of the metastasis. A planning organ at risk volume (PRV) expansion of 2 mm will be added for organs at risk (OAR) and dose constraints apply to this PRV. It is strongly recommended that dose constraints not be exceeded. If a dose constraint cannot be achieved due to overlap of the target with an organ at risk or its PRV, the fractionation can be increased or the target coverage compromised in order to meet the constraint. Treatment will be prescribed to the periphery of the target (80% of the dose) covering the 90% of the PTV. Dose constraints of organs at risk will be in accordance with the recommendations of the American Association of Physicist in Medicine (AAPM) task group 101 report [26].

#### Pembrolizumab

Pembrolizumab will be administered intravenously at a fixed dose of 200 mg per cycle. Pembrolizumab will be continued for up to 2 years until clinical progression or unacceptable toxicity. Pembrolizumab will be continued in clinically stable patients with initial evidence of disease progression. For suspected immune-related adverse reactions, adequate evaluation to confirm etiology or exclude other causes should be ensured. Based on the severity of the adverse reaction, pembrolizumab should be withheld and corticosteroids administered. Upon improvement to grade ≤1, corticosteroid taper should be initiated and continued over at least 1 month. Pembrolizumab may be restarted within 12 weeks after last dose of pembrolizumab if the adverse reaction remains at grade ≤1 and corticosteroid dose has been reduced to ≤10 mg prednisone or equivalent per day. Pembrolizumab must be permanently discontinued for any grade 3 immune related adverse reaction that recurs and for any grade 4 immune related adverse reaction toxicity, except for endocrinopathies that are controlled with replacement hormones. The subject may also discontinue protocol in case of intercurrent illness which would in the judgment of the investigator affect patient safety, the ability to deliver treatment or by request of the patient.

Subjects who stop pembrolizumab with stable disease or better may be eligible for up to 1 year of additional pembrolizumab therapy if they progress after stopping study treatment (only if the study remains open and the subject meets certain criteria).

#### Evaluation of the immunological response

The study requires blood samples (ethylenediaminetetraacetic acid (EDTA) and serum) before start of anti-PD-1 treatment, before start of SBRT, 7 days after end of radiotherapy and at the time of evaluation. The samples will be analysed with FACS phenotyping, UPLC and ELISA. The immune response will be analysed with a comprehensive immunophenotyping on peripheral blood, looking at absolute lymphocyte count, absolute neutrophil count/absolute lymphocyte count, serum tryptophan and kynurenine, C-reactive protein and cytokines, serum vascular endothelial growth factor levels, frequencies of Foxp3+ regulatory T cells, indoleamine 2, 3-dioxygenase+ plasmacytoid dendritic cells and myeloid derived suppressor cells, next to functional analysis looking at shifts in Th1/Th2/Th17 polarization as a function of treatment [27, 28].

#### Concomitant care

In general, medications or vaccinations specifically prohibited in the exclusion criteria are not allowed during the ongoing trial. Systemic glucocorticoids may be administered to modulate symptoms from an event of suspected immunologic etiology. All treatments that the investigator considers necessary for a subject’s welfare may be administered at the discretion of the investigator in keeping with the community standards of medical care. Adverse events will be managed according to most recent available guidelines.

### Follow-up

Patients will be seen before the start of each treatment cycle during the whole course of pembrolizumab therapy. At each visit, a history, directed physical examination and a routine laboratory blood analysis will be conducted with recording of the toxicity. Tumors will be re-evaluated every 9 weeks. A safety follow-up visit will find place approximately 30 days after the last dose of trial treatment or before the initiation of a new anti-cancer treatment, whichever comes first. Once a subject experiences confirmed disease progression or starts a new anti-cancer therapy, the subject moves into the survival follow-up phase and should be contacted by telephone every 12 weeks to assess for survival status until death, withdrawal of consent, or the end of the study, whichever occurs first. Subjects who discontinue trial treatment for a reason other than disease progression will move into the follow-up phase and will be assessed every 12 weeks by radiologic imaging to monitor disease status.

### Sample size

In the first phase of the trial 20 patients will be recruited and randomly assigned to a treatment arm. If the chance that the posterior toxicity probability rate exceeds 0.2 is more than 0.7 in a certain arm, this arm will be closed.

The secondary endpoint is the assessment of the response rate of the combination treatment in non-irradiated metastases in every arm. For this analysis a Simon’s two-stage optimum design will be used [29]. The arm with the best response rate and with acceptable toxicity will be used to proceed into the second phase. The null hypothesis that the true response rate is 0.21 [10] will be tested against a one-sided alternative. If there are two or fewer responses, the alternative hypothesis will be rejected. Otherwise 13 additional patients will be accrued for a phase II trial. The null hypothesis will be rejected if 7 or more responses are observed in 23 patients. This design yields a type I error rate of 0.15 and power of 0.8 when the true response rate is 0.41 (Table 2). We consider the chance that both arms in phase 1 are deemed safe with equal toxicity and equal response rates very small. Since both arms will then qualify to continue to phase 2, selection of one arm will depend on secondary outcomes available at that time (e.g. PFS, OS).

**Table 2:** Simon’s two-stage optimum design

Optimal two stage design	Optimum design
First stage sample size (n1)	10
r1	2
Maximum sample size (n)	23
r2	6

### Data analysis

- The current trial aims to evaluate the safety of the combination treatment of pembrolizumab with SBRT and determine whether the sequence of these therapies matters. The primary endpoint is DLT assessed using CTCAE version 4.0. The SBRT schedule associated with DLT in <20% of patients will be determined based on the incidence of treatment-related adverse events.
- Response rates will be determined using RECIST v1.1. The null hypothesis that the true response rate is 0.21 [10] will be tested against a one-sided alternative. The null hypothesis will be rejected if seven or more responses are observed in 23 patients.
- Survival times are defined from the day of randomization until progression, last follow-up or death. Cases will be censored at last follow up visit if no progression was observed. Multivariate analysis will be performed according to the cox-regression method.
- For the evaluation of immunological markers, median values between two groups will be compared by the Mann–Whitney U test, between >2 groups with Kruskal–Wallis testing. To compare proportions of categorical variables, the Pearson’s Chi^2^ test or Fisher’s Exact test will be used. To evaluate correlations, Spearman correlation coefficients will be calculated. All statistical analyses will be performed using SPSS 24.0 (SPSS Inc, Chicago, IL, USA), a *P* value <0.05 will be considered statistically significant.
- When missing data occurs, analysis will find place with the available data. Due to the small sample size, no imputing will find place.

### Study approval and recruitment

This trial is approved by the Ethics committee of the Ghent University Hospital (EC2016/0661) and is registered on clinicaltrials.gov (NCT02826564).

Patient recruitment finds place at the Ghent University Hospital, Belgium. The first participant was enrolled on November 14th 2016. Participants are currently being recruited and enrolled.

Protocol version 3.0; 12th of April, 2017.

### Monitoring

- Safety analysis will find place when all participants have passed 12 weeks after administration of SBRT. Immediately before allocation of a new participant, the posterior toxicity probability will be calculated by the statisticians in order to evaluate whether an arm should be terminated. Unless an arm is closed, only the statisticians will have access to the data regarding posterior toxicity probability.
- The trial conduct is monitored at least once annually by Bimetra Clinical Research Center Ghent and by MSD Belgium. This is independent from the investigators and the sponsor.

## Discussion

Immunotherapy has lead to breakthroughs in various solid tumors and can induce spectacular clinical responses. Yet unfortunately, responses are still only seen in the minority of patients. Several pre-clinical and clinical evidence indicate that radiotherapy instigates a systemic anti-cancer immune effect. One of these immune effects is the upregulation of PD-L1 expression and the presence of TILs in the tumor microenvironment. Therefore, the hypothesis was formed that the combination of anti-PD1 treatment with radiotherapy could work synergistically and raise response rates in patients with metastatic urothelial carcinoma. The current phase I/II trial will, firstly, investigate the safety of two different sequences of this combination treatment. In a second phase, the response rates of the combination treatment will be assessed. Throughout the study, the systemic immune effect of both treatments will be monitored on different time points. This information can aid in the fine tuning of combination therapies and may identify predictors of response or resistance to therapy or even pinpoint new potential targets for immunotherapy.

## Additional file

**Additional file 1: Table S1.** Schedule of enrolment, interventions and assessments.

## Abbreviations

AAPM: American Association of Physicist in Medicine
aPTT: activated partial thromboplastin time
CTCAE: Common Terminology Criteria for Adverse Events
DLT: dose limiting toxicity
EDTA: ethylenediaminetetraacetic acid
ELISA: enzyme-linked immunesorbent assay
FACS: fluorescence-activated cell sorting
GTV: gross target volume
INR: international normalized ratio
irRC: immune related response criteria
OAR: organs at risk
OS: overall survival
PRV: planning organ at risk volume
PFS: progression-free survival
PT: prothrombin time
PTV: planning target volume
RECIST: response evaluation criteria in solid tumors
SBRT: stereotactic body radiotherapy
TITE-CRM: time-to-event continual reassessment method
ULN: upper limit of normal
