# Supplementary material for: A phase I/II trial of fixed-dose stereotactic body radiotherapy with sequential or concurrent pembrolizumab in metastatic urothelial carcinoma: evaluation of safety and clinical and immunologic response
Source: J Transl Med. 2017 Jun 29;15:150. doi: 10.1186/s12967-017-1251-3 (PMC5492401; doi:10.1186/s12967-017-1251-3)
Supplement: Supplementary file 1 — Additional file 1: Table S1. Schedule of enrolment, interventions and assessments. [file 12967_2017_1251_MOESM1_ESM.docx]

**Additional table**

Table S1. Schedule of enrolment, interventions and assessments

| **Trial Period:** | | | | | **Screening Phase** | |  | |  | | **Study Treatment** | | | | | | | | | | | | | | **Post-Treatment** | |
| --- | --- | --- | --- | --- | --- | --- | --- | --- | --- | --- | --- | --- | --- | --- | --- | --- | --- | --- | --- | --- | --- | --- | --- | --- | --- | --- |
| Treatment Cycle/Title: | | | | | Pre-screening (Visit 1) | Main Study Screening (Visit 2) | SBRT | | | | | 1 | |  | 2 | SBRT | | | 3 | |  | | 4 | safety evaluation | Safety Follow-up | Survival Follow-up |
| Scheduling Window (Days): | | | | |  | -28 to -5 | -5 | -3 | | -1 | | 1 | | 7 | 22 | 38 | 40 | 42 | 43 | | 49 | | 64 | A: d84 B: d126 | 30 days post discon. | Every 12 weeks |
|  |  | **General assessments** | | | | | | | | | | | | | | | | | | | | | | | | |
| Pre-screening Consent | | | | | ● |  |  |  | |  | |  |  | |  |  |  |  |  | |  | |  |  |  |  |
| Informed Consent | | | | |  | ● |  |  | |  | |  |  | |  |  |  |  |  | |  | |  |  |  |  |
| Inclusion/Exclusion Criteria | | | | |  | ● |  |  | |  | |  |  | |  |  |  |  |  | |  | |  |  |  |  |
| Demographics and Medical History | | | | |  | ● |  |  | |  | |  |  | |  |  |  |  |  | |  | |  |  |  |  |
| Prior and Concomitant Medication Review | | | | |  | ● |  |  | |  | |  |  | |  |  |  |  |  | |  | |  |  |  |  |
| Treatment Allocation | | | | |  | ● |  |  | |  | |  |  | |  |  |  |  |  | |  | |  |  |  |  |
| Post-study anticancer therapy status | | | | |  |  |  |  | |  | |  |  | |  |  |  |  |  | |  | |  |  |  |  |
| Survival Status | | | | |  |  |  |  | |  | |  |  | |  |  |  |  |  | |  | |  |  |  | ● |
|  |  | **Trial Interventions** | | | | | | | | | | | | | | | | | | | | | | | | |
| Pembrolizumab | | | | |  |  |  |  | |  | | ● |  | | ● |  |  |  | ● | |  | | ● |  |  |  |
| SBRT | | | Arm A | |  |  | ● | ● | | ● | |  |  | |  |  |  |  |  | |  | |  |  |  |  |
|  |  |  | Arm B | |  |  |  |  | |  | |  |  | |  | ● | ● | ● |  | |  | |  |  |  |  |
|  |  | **Clinical Procedures/Assessments** | | | | | | | | | | | | | | | | | | | | | | | | |
| Review Adverse Events | | | | |  |  | ● |  | |  | |  |  | | ● |  |  |  | ● | |  | ● | |  | ● |  |
| Full Physical Examination | | | | |  | ● |  |  | |  | |  |  | |  |  |  |  |  | |  |  | | ● | ● |  |
| Directed Physical Examination | | | | |  |  | ● |  | |  | | ● |  | | ● |  |  |  | ● | |  | ● | |  |  |  |
| Vital Signs and Weight | | | | |  | ● | ● |  | |  | | ● |  | | ● |  |  |  | ● | |  | ● | | ● | ● |  |
| ECOG Performance Status | | | | |  | ● | ● |  | |  | | ● |  | |  |  |  |  | ● | |  |  | | ● |  |  |
|  |  | **Laboratory Procedures/Assessments: analysis performed by local laboratory** | | | | | | | | | | | | | | | | | | | | | | | | |
| Pregnancy Test | | | | |  | ● |  |  | |  | |  |  | |  |  |  |  |  |  | |  | |  |  |  |
| Laboratory blood analysis | | | | |  | ● |  |  | |  | | ● |  | | ● |  |  |  | ● |  | | ● | | ● |  |  |
| Urinalysis | | | | |  | ● |  |  | |  | | ● |  | | ● |  |  |  | ● |  | | ● | | ● | ● |  |
|  |  | **Efficacy Measurements** | | | | | | | | | | | | | | | | | | | | | | | | |
| Tumor Imaging | | | | | ● |  |  |  | |  | |  |  | |  |  |  |  |  |  | |  | | ● |  |  |
|  |  | **Tumor Biopsies/Archival Tissue Collection/Correlative Studies Blood** | | | | | | | | | | | | | | | | | | | | | | | | |
| Archival or Newly Obtained Tissue Collection | | | | |  | ● |  |  | |  | |  |  | |  |  |  |  |  |  | |  | |  |  |  |
| Correlative Studies: Blood Collection | | | | Arm A |  |  | ● |  | |  | |  | ● | |  |  |  |  |  |  | |  | | ● |  |  |
|  |  |  |  | Arm B |  |  |  |  | |  | |  | ● | |  | ● |  |  |  |  | |  | | ● |  |  |
